# Clinical significance of CD151 overexpression in subtypes of invasive breast cancer

**DOI:** 10.1038/bjc.2012.11

**Published:** 2012-01-31

**Authors:** M J Kwon, S Park, J Y Choi, E Oh, Y J Kim, Y-H Park, E Y Cho, M J Kwon, S J Nam, Y-H Im, Y K Shin, Y-L Choi

**Affiliations:** 1 Advanced Institutes of Convergence Technology, Suwon, Gyeonggi-do, Korea; 2 Division of Medical Oncology, Department of Internal Medicine, Seoul St. Mary's Hospital, Catholic University, Seoul, Korea; 3 Laboratory of Molecular Pathology and Cancer Genomics, Department of Pharmacy, College of Pharmacy, Seoul National University, Seoul, Korea; 4 Research Institute of Pharmaceutical Science, College of Pharmacy, Seoul National University, Seoul, Korea; 5 Laboratory of Cancer Genomics and Molecular Pathology, Samsung Biomedical Research Institute, Samsung Medical Center, Seoul, Korea; 6 Division of Hematology/Oncology, Department of Medicine, Samsung Medical Center, Sungkyunkwan University School of Medicine, Seoul, Korea; 7 Department of Pathology, Samsung Medical Center, Sungkyunkwan University School of Medicine, Irwon-ro 81, Gangnam-gu, Seoul 135-710, Korea; 8 Department of Pathology, Hallym University Sacred Heart Hospital, Hallym University College of Medicine, Anyang, Korea; 9 Division of Breast and Endocrine Surgery, Department of Surgery, Samsung Medical Center, Sungkyunkwan University School of Medicine, Seoul, Korea

**Keywords:** CD151, breast cancer, five subtypes, prognosis, tetraspanin

## Abstract

**Background:**

CD151 is a member of the tetraspanin family, which interacts with laminin-binding integrins and other tetraspanins. This protein is implicated in motility, invasion, and metastasis of cancer cells, but the prevalence of CD151 expression in subtypes of breast cancers and its influence on clinical outcome remains to be evaluated.

**Methods and results:**

The immunohistochemistry-based tissue microarray analysis showed that 127 (14.3%) cases overexpressed CD151 among 886 breast cancer patients. CD151 overexpression was found to be significantly associated with larger tumour size, higher nodal stage, advanced stage, absence of oestrogen receptor and progesterone receptor, and human epidermal growth factor receptor 2 overexpression. CD151 overexpression resulted in poorer overall survival (OS) (*P*<0.001) and disease-free survival (*P*=0.02), and stage II and III patients with CD151 overexpression demonstrated substantially poorer OS (*P*=0.0474 and 0.0169). In the five subtypes analyses, CD151 overexpression retained its adverse impact on OS in the Luminal A (*P*=0.0105) and quintuple-negative breast cancer (QNBC) subtypes, one subgroup of triple-negative breast cancer (*P*=0.0170). Multivariate analysis that included stage, subtype, and adjuvant chemotherapy showed that CD151 overexpression was independently associated with poor OS in invasive breast cancer.

**Conclusion:**

CD151 overexpression may be a potential molecular therapeutic target for breast cancer, especially in QNBC subtype and more advanced stages of breast cancer.

CD151 is a member of the mammalian tetraspanins, which are transmembrane proteins involved in a variety of biological processes including the immune system, fertilisation, infectious processes, and tumour progression (Maecker *et al*, 1997; Hemler, 2008). Tetraspanin proteins form complexes between themselves or with other non-tetraspanin molecules such as integrins, immunoglobulin superfamily members, and signalling molecules, and carry out several functions depending on interacting partners (Hemler, 2005; Zoller, 2009). Particularly, CD151 contributes to integrin-dependent cell adhesion and motility by directly interacting with laminin-binding integrins (*α*_3_*β*_1_, *α*_6_*β*_1_, *α*_6_*β*_4_, and *α*_7_*β*_1_) (Hemler, 2008). A recent study also reported that CD151 has a role in proliferation of mammalian epithelial cells, suggesting that CD151 may contribute to the tumour cell growth (Novitskaya *et al*, 2010). Tetraspanin CD151 is expressed in most of cells and tissue types showing high expression in epithelial and endothelial cells (Sincock *et al*, 1997; Zoller, 2009). Deregulation of several tetraspanins is observed in human cancer, and these upregulation or downregulation are of clinical significance in some malignancies (Romanska and Berditchevski, 2011). Upregulation of CD151 is found in many tumour types and CD151 overexpression was associated with poor prognosis in non-small cell lung (Tokuhara *et al*, 2001), colon cancer (Hashida *et al*, 2003), hepatocellular (Ke *et al*, 2009), pancreatic (Zhu *et al*, 2011), oesophageal (Suzuki *et al*, 2010), and endometrial cancer (Voss *et al*, 2011). In addition, there have been several evidences supporting the contribution of CD151 in tumour progression. Although tetraspanins CD82 and CD9 are known to suppress metastasis (Zoller, 2009), CD151 promotes metastasis by regulating tumour cell migration (Zijlstra *et al*, 2008). Specifically, overexpression of CD151 enhances cell motility, invasion, and metastasis in colon cancer and fibrosarcoma cells (Kohno *et al*, 2002). In hepatocelluar carcinoma cells, CD151 expression promotes invasiveness of tumour cells in association with induction of epithelial–mesenchymal transitions (Ke *et al*, 2011).

Based on the association of *α*_6_*β*_4_ integrin in mammary tumourigenesis (Shaw *et al*, 1997; Zahir *et al*, 2003; Guo *et al*, 2006), the relevance of CD151 in breast cancer was also hypothesised. Indeed, Yang *et al* (2008) showed that CD151 expression is elevated in breast cancer, with even more upregulation in high-grade and oestrogen-negative subtypes including basal-like breast cancer. Moreover, it was demonstrated that loss of CD151 decreased the integrin-mediated cell migration, spreading, invasion, and signalling (through FAK, Rac1, and lck) of basal-like mammary cell lines with the effect on the subcellular distribution of *α*_6_ integrins (Yang *et al*, 2008). The delayed breast cancer progression by CD151 ablation was also shown in mouse xenograft models established using basal-like cell line, suggesting that CD151 may be a novel therapeutic target in certain breast cancer subtypes (Yang *et al*, 2008). High expression of CD151 in high-grade breast cancer was also confirmed in the recent study by Sadej *et al* (2009). Furthermore, in the same study, CD151 overexpression was shown to correlate with decreased survival of patients with breast cancer when assessed in 56 cases (Sadej *et al*, 2009). However, the association of CD151 expression with clinical outcome as well as its significance as prognostic factor in breast cancer patients is still unclear. Moreover, a systematic approach examining the incidence of CD151 expression and the significance of CD151 on clinical outcomes in breast cancer subtypes has not been undertaken. In order to select the appropriate breast cancer patients for targeted therapy, a detailed analysis using marker-driven subtyping in patient populations is critical. Therefore, to define the prognostic impact of CD151 expression in breast cancer subtypes, we divided 886 patients with breast cancer into five subtypes and assessed the relationship of CD151 expression with clinical outcome including overall survival (OS) and disease-free progression survival in each subtype.

## Materials and Methods

### Study samples and five subtypes information

A tissue microarray (TMA) constructed from duplicate 2 mm cores of invasive breast carcinomas from 1290 primary invasive breast cancer samples was utilised for the analysis of CD151 status. This retrospective cohort was named ‘Samsung Medical Center Breast Cancer Biomarker Study' and was originally intended for the clinical validation of a novel biomarker set according to the breast cancer subtype (Choi *et al*, 2010). The clinical features of this cohort were as follows: (1) patients did not receive cytotoxic chemotherapy or hormones before surgery; (2) all oestrogen receptor (ER)-positive patients underwent hormonal therapy with tamoxifen; (3) none of the patients underwent anti-human epidermal growth factor receptor 2 (HER2) therapy. The pathological tumour stage was assessed according to the American Joint Committee on Cancer (AJCC) 6 Staging System. The histological grade was determined according to the Bloom–Richardson classification scheme. This study was approved by the Institutional Review Board at the Samsung Medical Center (Seoul, Korea) in accordance with the Declaration of Helsinki.

Only 951 of the cases had subtype information (Choi *et al*, 2010). Each case was divided into five subgroups according to the status of ER, progesterone receptor (PR), HER2, and basal markers (either epidermal growth factor receptor (EGFR) or cytokeratin 5/6 (CK5/6)) as described previously (Choi *et al*, 2010): (1) Luminal A (ER+ or PR+/HER2−), (2) Luminal B (ER+ or PR+/HER2+), (3) HER2 (ER−/PR−/HER2+), triple-negative breast cancer (TNBC, ER−/PR−/HER2−), TNBC subtype was further divided into (4) basal-like breast cancer (BLBC, ER−/PR−/HER2−/EGFR+ or CK5/6+), and (5) quintuple-negative breast cancer (QNBC, ER−/PR−/HER2−/EGFR−/CK5/6−) (Choi *et al*, 2010). Although the ER and PR status were acquired from the pathological report using the semi-quantitative Allred score, the HER2, CK5/6, and EGFR status were determined from the TMA analysis. Tumours were classified as HER2 positive if they had a score of 3+ in regard to the staining on IHC and/or gene amplification as determined by fluorescence *in situ* hybridisation when using HER2, such that the chromosome 17 ratio was >2.2. Cytokeratin 5/6 was interpreted as positive if there was any observation of cytoplasm and/or membranous staining. The EGFR status was scored as positive when at least 10% of the tumour cells showed strong membranous staining.

### Immunohistochemical analysis

Immunohistochemical analyses were performed on the paraffin sections as described previously (Chien *et al*, 2008). The TMA sections were incubated with the monoclonal mouse anti-human CD151 antibody at room temperature for 60 min (1 : 100 dilution, RLM30, Novocastra, Newcastle upon Tyne, UK). Specimens were then incubated with a 1 : 1000 dilution of biotinylated goat anti-mouse IgG (Vector Laboratories, Burlingame, CA, USA) for 1 h at room temperature after washing with PBS. CD151 expression was scored using the HER2 semi-quantitative method based on the following four classes (Wolff *et al*, 2007): score 0 (no staining is observed or cell membrane staining is observed in <10% of the tumour cells), score 1+ (a faint perceptible membrane staining can be detected in >10% of the tumour cells. The cells are only stained in parts of their membrane), score 2+ (a weak to moderate complete membrane staining is observed in >10% of the tumour cells), and score 3+ (a strong complete membrane staining is observed in >30% of the tumour cells). Scores ranging from 0 to 2+ were classified as CD151-low expression and cases that had a score of 3+ were classified into the CD151-high expression group. Two pathologists (MJK and YLC) independently scored the immunohistochemical staining and were blinded with respect to the results of the other markers and the outcome data.

### Statistical analysis

Disease-free survival (DFS) was defined as the time from the date of diagnosis to the date of documented relapse, including locoregional recurrence and distant metastasis. Overall survival was expressed as the number of months from diagnosis of breast cancer to the date of death. Differences in the frequencies of the basic characteristics, clinical parameters, and subtypes were statistically analysed using either the chi-square test or the Fisher's exact test in cases when the expected values of any of the cells were <5. Survival curves were constructed using the Kaplan–Meier method and the log-rank test was used to compare the mean survival rates across the groups. The log-rank test with Bonferroni's correction was used for the subgroup survival analysis. For the multivariate analysis, Cox regression models were constructed in order to estimate the adjusted hazard ratios (HRs) of the groups according to stage, adjuvant chemotherapy, and subtype. *P*-values <0.05 were considered to be statistically significant and all of the *P*-values corresponded to two-sided significance tests. All of the statistical analyses were performed using SPSS 16.0 (Chicago, IL, USA). The ‘REMARK’ criteria of the National Cancer Institute was used in the design, analysis, and interpretation of the results (McShane *et al*, 2005).

## Results

### Patient characteristics and CD151 expression

**Figure 1:**
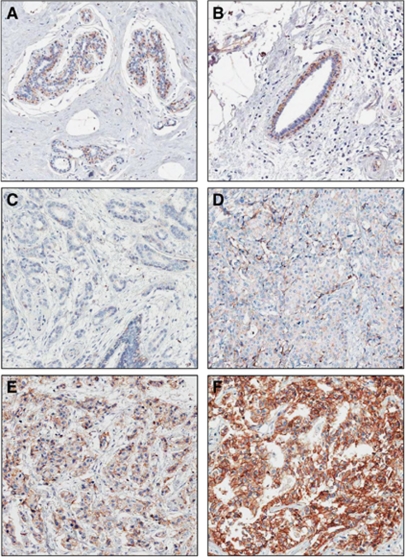
CD151 expression in normal tubule-lobular unit (**A**) and duct (**B**) in breast tissue ( × 200). CD151 expression is localised to the cytoplasm of the basal layer. Representative cases of each score of CD151 in invasive breast cancer (**C**, score 0; **D**, score 1; **E**, score 2; **F**, score 3, × 200). The strong membranous overexpression of CD151 is noted in invasive breast cancer (**F**).

In 65 cases, CD151 stain was considered to be unsatisfactory because of loss of tissue core or no invasive cancer component, and these cases were further excluded from 951 cases with subtype information. Therefore, a total of 886 cases with informative immunohistochemical results were included in this analysis. All of the patients were Korean females who had curative resection of their primary tumours and axillary node dissection or sentinel node sampling. The median age at diagnosis was 46 years (range, 23–80 years). The characteristics of the patients are provided in Table 1. In the normal breast tissue, CD151 was expressed in the basal-myoepithelial cell layer surrounding both ducts and tubule-lobular units (Figures 1A and B). The invasive cancers showed CD151 expression predominantly localised to the membrane, with expression occurring in the cytoplasm in some cases (Figures 1D, E and F). The numbers of patients in each group of CD151 expression were as follows: score 0, 80 (9.0%); score 1, 356 (40.2%); score 2, 323 (36.5%); and score 3, 127 (14.3%). In all, 127 (14.3%) cases were identified as CD151-high expression and 759 (85.7%) cases were classified as CD151-low expression. CD151 overexpression was significantly associated with a more advanced stage (*P*<0.001), larger tumour size (*P*<0.001), lymph node involvement (*P*<0.001), and absence of ER (*P*<0.001) and PR (*P*=0.009) (Table 1). There were no significant differences in the distribution of adjuvant chemotherapy modalities between the CD151-low group and the CD151-high group (*P*=0.409). CD151 overexpression was detected more frequently in breast cancers with HER2 overexpression (21.9%) than in HER2-negative breast cancers (11.8%, *P*<0.001). When CD151 overexpression was compared among the breast cancer subtypes (Luminal A, Luminal B, HER2, BLBC, and QNBC), CD151 overexpression varied significantly according to the breast cancer subtype (*P*<0.001). The Luminal A subtype had a lower incidence in tumours with CD151 expression. CD151 overexpression was most frequent in the HER2 subtype (27.4%) (Table 1).

### Impact of CD151 overexpression on survival in breast cancer according to stage and subtype

**Figure 2:**
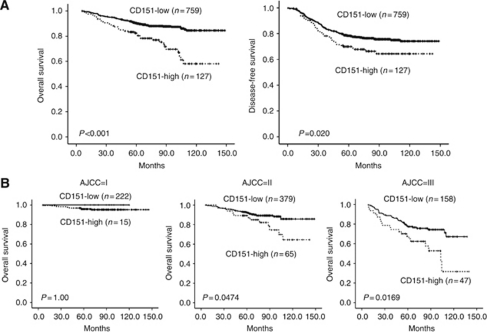
(**A**) Overall survival and DFS according to CD151 expression in breast cancer. (**B**) The impact of CD151 expression in breast cancer on OS according to AJCC stage.

**Figure 3:**
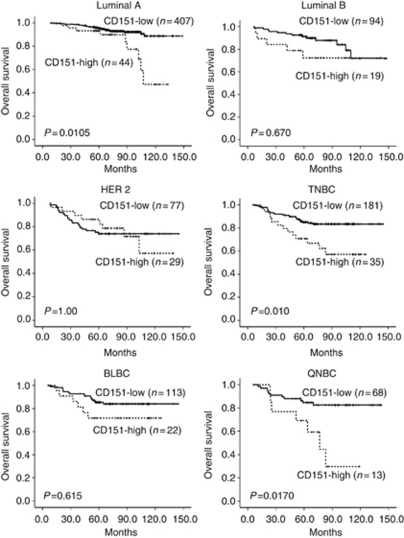
The impact of CD151 expression in breast cancer on OS according to five subtypes. TNBC was subclassified into BLBC and QNBC.

The time of DFS ranged from 0 to 148.7 months with a median of 68.8 months. During the study period, 24.6% of the women (218 out of 886) had local recurrence and/or metastasis. Duration of OS ranged from 6.0 to 148.7 months with a median of 74.9 months. During the study period, 14.1% of women (125 out of 886) died, whereas the remaining 761 were still alive at the end of the study. The breast cancer patients with CD151 overexpression demonstrated substantially poorer OS (CD151-high *vs* CD151-low; 109.8 months (95% confidence interval (CI), 100.9–118.7 months) *vs* 134.1 months (95% CI, 131.3–137.0 months), *P*<0.001) and DFS (CD151-high *vs* CD151-low; 104.2 months (95% CI, 94.6–113.7 months) *vs* 120.0 months (95% CI, 116.2–123.7 months), *P*=0.020) (Figure 2A). We performed survival analyses according to the AJCC stage of the breast cancer. Although CD151 overexpression did not show any impact on survival in regard to AJCC stage I cancer, CD151 overexpression had a significant influence on OS in stage II cancer (CD151-high *vs* CD151-low, 117.3 months (95% CI, 106.2–128.4 months) *vs* 135.6 months (95% CI, 131.7–139.5 months), *P*=0.0474) and stage III cancer (CD151-high *vs* CD1515-low, 86.7 months (95% CI, 70.9–102.5 months) *vs* 110.4 months (103.1–117.7 months), *P*=0.0169) (Figure 2B). We also performed subgroup analyses according to breast cancer subtype. CD151 overexpression did not markedly influence OS in the Luminal B or HER2 subtypes (Figure 3). Luminal A subtype with CD151 overexpression showed a significantly poor OS (CD151-high *vs* CD151-low, 109.4 months (95% CI, 97.5–121.4) *vs* 139.6 months (95% CI, 136.2–143.0); *P*=0.0105) (Figure 3). The TNBC subtype with CD151 overexpression had a more rapid deteriorating clinical course (median OS, 91.6 months (95% CI, 76.8–106.5)) compared with that of CD151-low patients with TNBCs (median OS, 126.9 months (95% CI, 120.9–133.0)), in terms of OS (*P*=0.010) (Figure 3).

Next, the prognostic value of CD151 expression was evaluated in the subgroup analyses according to five subtypes as the QNBC subtype was expected to be insensitive to chemotherapy. CD151 overexpression did not significantly affect OS in BLBCs with CD151 overexpression, but a trend toward poorer OS did exist (CD151-high *vs* CD151-low, 99.7 months (95% CI, 81.5–117.8) *vs* 127.7 months (95% CI, 120.2–135.2); *P*=0.615). In regard to the QNBC subtype, CD151 overexpression retained its significant adverse impact on OS (CD151-high *vs* CD151-low, 74.9 months (95% CI, 54.0–95.7) *vs* 123.7 months (95% CI, 113.6–133.8); *P*=0.0170) (Figure 3).

### Prognostic factor analyses

The factors that predicted poor OS based on the univariate analysis were CD151 overexpression, AJCC stage, cancer subtype, and adjuvant chemotherapy (Table 2). CD151 overexpression, AJCC stage, and subtype were significant prognostic factors for DFS. In the Cox regression model, the prognostic factors for OS in all of the patients were AJCC stage, breast cancer subtype, adjuvant chemotherapy, and CD151 overexpression. In regard to the DFS, only the AJCC stage retained its statistical significance at the multivariate level (Table 3). Breast cancer patients with CD151 overexpression demonstrated a substantially lower OS with a 1.65-fold (*P*=0.034; HR, 1.65; 95% CI, 1.03–2.59) higher risk of death after adjusting for AJCC stage, breast subtype, and adjuvant chemotherapy (Table 3).

## Discussion

The detailed clinical significance of CD151 overexpression in a large cohort of patients with breast cancer has not been previously reported. In this study of 886 breast cancer cases, we found that CD151 overexpression is an independent negative predictor of OS in patients with breast cancer and its worse impact on OS was retained in Luminal A and QBNC subtypes.

The normal expression of CD151 in the breast tissue was limited to the basal-myoepithelial cell layer surrounding both ducts and lobular alveolae, which agrees with the findings of a previous report (Yang *et al*, 2008). By contrast, variable patterns of CD151 expression were seen in the invasive breast cancer tissues, which ranged from absence to diffuse, strong overexpression occurred mainly in the membrane and/or cytoplasm of the tumour cells. Yang *et al* (2008) have detected the significant associations between CD151 expression and tumour grade, ER status, and combination of ER/HER2 status in 124 breast cancer cases. This is consistent with our findings because the ER-negative breast cancers, which had a higher proportion of CD151 overexpression, contained both HER2 and TNBC subtypes. Our data also indicated that the HER2 subtype had elevated CD151 expression, and that the Luminal A subtype had the lowest proportion of CD151 expression. However, they did not examine the long-term outcome or recurrence in their study, and they had calculated the CD151 positivity as 31% (Yang *et al*, 2008), whereas we calculated it as 14.3% in our study. These differences in the cutoff values to identify positive cases may be controversial. However, the clinicopathological characteristics of CD151 overexpression cases were similar in both cohorts.

On the other hand, it was found in a previous study by Sadej *et al* (2009) that CD151 overexpression in breast cancers is associated with decreased OS based on 56 cases of breast invasive ductal carcinomas, 30.4% of which were classified as being CD151 positive. Furthermore, they have shown that CD151 expression is also positively associated with the involvement of regional lymph nodes. However, there were no associations between CD151 expression and ER status, tumour grade, disease stage, and age. Compared with these two previous studies of CD151 in breast cancer, our study utilised a larger number of cases that had sufficient follow-up data, including subtype analysis and thus this might be a reason for the discrepancy in results between this study and previous studies.

The upregulation of CD151 expression has been seen in many types of tumours and is generally associated with a poor prognosis (Romanska and Berditchevski, 2011). The positive rate or proportion of CD151 overexpression that is detectable by immunohistochemistry in other cancers is variable (Table 4). Furthermore, there is no consensus for the cutoff criteria of CD151 expression. We used the scoring method of HER2 in a semi-quantitative manner and cases with a score of 3+ were considered to have CD151-high expression. CD151 overexpression occurred mainly in the membrane and/or cytoplasm in the tumour cells of the cases. As CD151 is a transmembrane protein, its functional localisation is believed to be the cellular membrane. Therefore, we did not include cases that showed only cytoplasmic expression, which was rare in our CD151-high expression group.

In agreement with poor prognosis of CD151 overexpression in several cancer types, CD151 is a metastasis-promoting tetraspanin protein. Actually, CD151-transfected cancer cell lines enhanced cell migration and invasion (Kohno *et al*, 2002; Ang *et al*, 2010; Ke *et al*, 2011). The involvement of CD151 through regulation of cell motility in metastasis was also demonstrated *in vivo* (Zijlstra *et al*, 2008). Additionally, anti-CD151 antibody treatment of high-CD151-expressing tumour cells decreased cell migration and metastasis (Testa *et al*, 1999). CD151-blocking antibody was reported to inhibit invasion and intravasation at the site of the primary tumour (Zijlstra *et al*, 2008). Moreover, a recent *in vivo* study showed that CD151-null mice have markedly diminished experimental lung metastasis (Sadej *et al*, 2010).

In our study, CD151 overexpression was also associated with poor prognosis of breast cancer patients in line with its clinical significance in other types of cancer. In particular, high-CD151 expression was significantly correlated with a larger tumour size, higher lymph node involvement, and advanced stage of invasive breast cancer. Its association with a larger tumour size in this study can be explained by the previous findings showing that CD151 has a positive role in breast tumour cell growth *in vivo*, whereas its downregulation causes an inhibition of tumour cell growth (Sadej *et al*, 2009). In addition, CD151 ablation was found to inhibit the migration, invasion, and spreading of breast cancer cells in relation with its effect on the subcellular distribution of integrins, suggesting the promoting role of CD151 in breast tumour progression (Yang *et al*, 2008). Furthermore, a recent study has shown that the depletion of CD151 attenuates pulmonary metastasis of breast cancer cells by regulating transforming growth factor *β* signalling (Sadej *et al*, 2010). These results may support the relevance of high-CD151 expression to a higher lymph node involvement and thereby advanced stage of invasive breast cancer in this study. Combination of these promoting effects of CD151 expression on breast cancer progression including tumour size and lymph node involvement may be responsible for a poor prognosis of breast cancer patients with high-CD151 expression.

However, CD151 overexpression was found to be an independent negative prognosis factor for OS but not for DFS of patients with breast cancer in this study. In regard to DFS, only the AJCC stage retained its statistical significance after adjustments for other prognostic factors including CD151 expression, subtypes, and adjuvant chemotherapy, suggesting that AJCC stage is a strong predictor of recurrence of breast cancer superior to other prognostic factors in this study. It is unclear why this difference in the effect of CD151 expression on the prediction of OS or DFS was observed. However, it is likely that CD151 expression influences the recurrence of breast cancer indirectly together with other variables, not in a direct manner, and thereby its effects on the prediction of DFS may not be as strong as AJCC stage even though high-CD151 expression was significantly correlated with recurrence of breast cancer patients in univariate analysis.

In this study, CD151 overexpression was found in 21.9% of the HER2-positive cases, which includes both HER2 and Luminal B subtypes, and was found in 27.4% of the HER2 subtype. Recently, it has been reported that CD151 is one of the mechanisms of resistance to anti-ErbB2 (HER2) agents, which suggests that targeting CD151 offers potential advantages such as drug sensitisation (Yang *et al*, 2010). Interestingly, although CD151 overexpression was observed most frequently in HER2 subtype, a significant effect on survival was not shown. However, we cannot make any definitive conclusions based on this result as our cohort did not receive anti-ErbB2 (HER2) therapy. The clinical significance of CD151 overexpression in the HER2 subtype should be investigated in a cohort that has received anti-ErbB2 (HER2) therapy. CD151-*α*_6_*β*_4_ integrin complexes may influence the sensitivity to ErbB2 (HER2)-targeted therapies as *α*_6_*β*_4_ enhances the signalling of ERBB family members (Guo *et al*, 2006). Constitutively activated proteins in these pathways may contribute to the clinical characteristics of HER2-positive tumours. Whether the CD151-associated HER2-positive breast cancer represents an independent disease entity remains unanswered and needs to be clarified in future studies. Thus, correlative analyses with CD151 and key proteins of these cascade pathways may be interesting to investigate so that the pathogenic role of CD151 in HER2-positive breast cancer is further clarified. CD151-high cases were rarest in the Luminal A subtype, but high-CD151 expression in this group was significantly associated with shorter OS. We also found that 16.2% of TNBC overexpressed CD151 and CD151-high cases showed a significantly poor OS consistent with the previous study demonstrating a role for CD151-*α*6 integrin complexes in basal-like breast cancer progression (Yang *et al*, 2008).

In conclusion, patients with CD151 expression have a more rapid deteriorating clinical course with poorer OS compared with those not expressing the protein. CD151 expression may be a potential molecular therapeutic target for breast cancer, especially in the Luminal A and QNBC subtypes, and advanced stages of cancers. Thus, more effective treatment should be adopted in this particular subset of patients by possibly administering CD151-targeted therapy during conventional chemotherapy and HER2-targeted therapy regimens.

## Figures and Tables

**Table 1:** Characteristics of patients with invasive breast cancer according to CD151 expression

			**CD151 expression**				
	**Number of patients**		**Low**		**High**		
**Characteristic**	***n*=886**	**(%)**	***n*=759 (85.7%)**	**(%)**	***n*=127 (14.3%)**	**(%)**	***P-*value**
*Age at diagnosis (years)*							
⩽ 35	33	3.7	30	90.9	3	9.1	0.61
>35	853	96.3	729	85.5	124	14.5	
							
*Tumour size*							**<0.001**
T1	363	41.0	330	90.9	33	9.1	
T2	462	52.1	384	83.1	78	16.9	
T3	61	6.9	45	73.8	16	26.2	
							
*Lymph node involvement*							**<0.001**
N0	469	52.9	420	89.6	49	10.4	
N1	228	25.7	195	85.5	33	14.5	
N2	109	12.3	79	72.5	30	27.5	
N3	80	9.0	65	81.3	15	18.8	
							
*AJCC stage*							**<0.001**
I	237	26.7	222	93.7	15	6.33	
II	444	50.1	379	85.4	65	14.6	
III	205	23.1	158	77.1	47	22.9	
							
*Oestrogen receptor*							**<0.001**
Negative	342	38.6	272	79.5	70	20.5	
Positive	544	61.4	487	89.5	57	10.5	
							
*Progesterone receptor*							**0.009**
Negative	485	54.7	402	82.9	83	17.1	
Positive	401	45.3	357	89.0	44	11.0	
							
*HER2*							**<0.001**
Negative	667	75.3	588	88.2	79	11.8	
Positive	219	24.7	171	78.1	48	21.9	
							
*Pathological type*							0.275
Ductal	812	91.6	691	85.1	121	14.9	
Lobular	25	2.8	22	88.0	3	12.0	
Others	49	5.5	46	93.9	3	6.12	
							
*Breast cancer subtype*							**<0.001**
Luminal A	451	50.9	407	90.2	44	9.8	
Luminal B	113	12.8	94	83.1	19	16.8	
HER2	106	12.0	77	72.6	29	27.4	
TNBC	216	24.4	181	83.8	35	16.2	
BLBC	135	15.2	113	83.8	22	16.2	
QNBC	81	9.1	68	84.0	13	16.0	
							
*CK5/6*							0.274
Negative	760	85.8	655	86.2	105	13.8	
Positive	126	14.2	104	82.5	22	17.5	
							
*EGFR*							0.336
Negative	762	86.0	649	85.2	113	14.8	
Positive	124	14.0	110	88.7	14	11.3	
							
*Adjuvant Chemotherapy*							0.409
No	148	16.7	130	87.8	18	12.2	
Chemotherapy	738	83.3	629	85.2	109	14.8	

Abbreviations: AJCC=American Joint Committee on Cancer; BLBC=basal-like breast cancer; HER2=human epidermal growth factor receptor 2; QNBC=quintuple-negative breast cancer; TNBC=triple-negative breast cancer. Statistically significant *P*-values (*P*<0.05) are shown in bold.

**Table 2:** Univariate analysis of the overall survival and progression-free survival in 886 patients with invasive breast cancer

	**Overall survival**		**Disease-free survival**	
**Prognostic factor**	**Number of events** **Hazard ratio (95% CI)**	***P*-value**	**Number of events** **Hazard ratio (95% CI)**	***P*-value**
*CD151 expression*				
Low	90/759		177/759	
High	35/127	**<0.001**	41/127	**0.021**
	2.50 (1.688-3.687)		1.50 (1.064-2.099)	
				
*AJCC stage*				
I	10/237		31/237	
II	54/444	**0.001**	93/444	**0.013**
	3.00 (1.526-5.883)		1.67 (1.114-2.512)	
III	61/205	**<0.001**	94/205	**<0.001**
	8.09 (4.416-15.79)		4.32 (2.879-6.489)	
				
*Subtype*				
Luminal A	38/451		102/451	
Luminal B	18/113	**0.017**	36/113	**0.020**
	1.98 (1.131-3.471)		1.57 (1.073-2.294)	
HER2	28/106	**<0.001**	33/106	**0.027**
	3.30 (2.020-5.376)		1.56 (1.053-2.309)	
TNBC	41/216	**<0.001**	47/216	0.858
	2.39 (1.533-3.712)		1.03 (0.730-1.458)	
				
*Adjuvant chemotherapy*				
No	33/148		30/148	
Yes	92/738	**0.009**	188/738	0.223
	0.59 (0.394-0.874)		1.27 (0.864-1.868)	

Abbreviations: AJCC=American Joint Committee on Cancer; CI=confidence interval; HER2=human epidermal growth factor receptor 2; TNBC=triple-negative breast cancer. Statistically significant *P*-values (*P*<0.05) are shown in bold.

**Table 3:** Multivariate analysis of the overall survival and progression-free survival in 886 patients with invasive breast cancer

	**Overall survival**		**Disease-free survival**	
**Prognostic factor**	**Hazard ratio (95% CI)**	***P*-value**	**Hazard ratio (95% CI)**	***P*-value**
*CD151 expression*				
Low				
High	1.65 (1.03-2.59)	**0.034**	1.15 (0.814-1.634)	0.421
				
*AJCC stage*				
I				
II	3.57 (1.791-7.103)	**<0.001**	1.63 (1.072-2.473)	**0.022**
III	11.5 (5.724-23.17)	**<0.001**	4.25 (2.766-6.531)	**<0.001**
				
*Subtype*				
Luminal A				
Luminal B	1.63 (0.926-2.868)	0.090	1.44 (0.980-2.109)	0.063
HER2	2.49 (1.518-4.091)	**<0.001**	1.40 (0.941-2.092)	0.097
TNBC	3.08 (1.969-4.815)	**<0.001**	1.18 (0.831-1.675)	0.356
				
*Adjuvant chemotherapy*				
No				
Yes	0.30 (0.195-0.451)	**<0.001**	0.88 (0.584-1.309)	0.514

Abbreviations: AJCC=American Joint Committee on Cancer; CI=confidence interval; HER2=human epidermal growth factor receptor 2; TNBC=triple-negative breast cancer. Statistically significant *P*-values (*P*<0.05) are shown in bold.

**Table 4:** CD151 expressions and positive rates in variable epithelial malignancy as measured by the immunohistochemical method

	**Country**	**Year**	**Organ**	**Cancer type**	**Number**	**Tissue**	**Antibody**	**Titer**	**Incubation**	**% positive**	**Cutoff of positive cases**	**Survival data**	**Multivariate data**
1. This study	Korea	2011	Breast	Invasive carcinoma	886	FFPE, TMA	RLM30, Novocastra	1 : 100	2 h, RT	14.6	3+ with diffuse complete membrane stating	Yes	Yes
2. Yang *et al* (2008)	USA	2008	Breast	Invasive ductal carcinoma	124	FFPE, TMA	RLM30, Novocastra	1 : 50	NA	31.0	2+ or 3+	None	None
3. Sadej *et al* (2009)	UK	2009	Breast	Invasive ductal carcinoma	56	FFPE	RLM30, Novocastra	1 : 50	NA	30.4	>10% of positive cells with weak to strong complete membrane staining	Yes	None
4. Voss *et al* (2011)	UK	2011	Uterus	Endometrial cancer	131	FFPE, TMA	RLM30, Novocastra	1 : 50	NA	58.7	>H-score 150	Yes	Yes
5. Yoo *et al* (2011)	Korea	2011	Kidney	Clear cell carcinoma	489	FFPE, TMA	RLM30, Novocastra	1 : 100	1 h, RT	47.5	>50% of positive cells with diffuse moderate or strong intensity	Yes	Yes
6. Suzuki *et al* (2010)	Japan	2010	Esophagus	Squamous cell carcinoma	138	FFPE	RLM30, Novocastra	1 : 50	4°C overnight	54.3	>10% of positive cells with weak to strong complete membrane staining	Yes	No
7. Huang *et al* (2010)	China	2010	Liver	Intrahepatic cholangiocarcinoma	140	FFPE, TMA	11G5a, Serotec	1 : 200	NA	53.6	>50% of tumour cells	Yes	Yes
8. Ke *et al* (2009)	China	2009	Liver	Hepatocellular carcinoma	520	FFPE, TMA	11G5a, Serotec	1 : 100	NA	59.8	>50% of tumour cells	Yes	Yes
9. Zhu *et al* (2011)	China	2011	Pancreas	Ductal adenocarcinoma	71	FFPE	sc-80715, Santa Cruz	1 : 00	1 h, RT	81.7	4–7 points, moderate to strong intensity	Yes	Yes
10. Ang *et al* (2004)	Australia	2004	Prostate	Adenocarcinoma	76	FFPE	11B1, purified IgG2a	4 ug ml^−1^	2 h, RT	23.0	>17.52 density and area measured by digitised image^a^	Yes	None
11. Hashida *et al* (2003)	Japan	2003	Colon	Adenocarcinoma	146	Frozen sections	SFA1.2B4	NA	2 h, RT	55.5	>120 multiplying of intensity and percentage of cells	Yes	Yes
12. Tokuhara *et al* (2001)	Japan	2001	Lung	Non-small cell carcinoma	145	Frozen sections	SFA1.2B4	NA	2 h, RT	54.5	>50% of tumour cells	Yes	Yes

Abbreviations: FFPE=formalin fixed paraffin embedded; NA=not available; RT=room temperature; TMA=tissue microarray.

Yes: The study contains negative correlation data between survival and CD151 overexpression; No: CD151 overexpression is not an independent factor for poor survival; None: the study does not contain survival data.

a Only cytoplasmic regions of the epithelial cell were measured.
